# Cytotoxicity of Labruscol, a New Resveratrol Dimer Produced by Grapevine Cell Suspensions, on Human Skin Melanoma Cancer Cell Line HT-144

**DOI:** 10.3390/molecules22111940

**Published:** 2017-11-09

**Authors:** Laetitia Nivelle, Jane Hubert, Eric Courot, Nicolas Borie, Jean-Hugues Renault, Jean-Marc Nuzillard, Dominique Harakat, Christophe Clément, Laurent Martiny, Dominique Delmas, Philippe Jeandet, Michel Tarpin

**Affiliations:** 1 Unité Matrice Extracellulaire et Dynamique Cellulaire, UMR CNRS 7369, SFR Cap-Santé FED 4231, UFR des Sciences Exactes et Naturelles, Université de Reims Champagne-Ardenne, BP 1039, 51687 Reims CEDEX 2, France; nivellelaetitia@gmail.com (L.N.); laurent.martiny@univ-reims.fr (L.M.); michel.tarpin@univ-reims.fr (M.T.); 2 Institut de Chimie Moléculaire de Reims, UMR CNRS 7312, SFR Cap-Santé FED 4231, UFR de Pharmacie, Université de Reims Champagne-Ardenne, 51687 Reims CEDEX 2, France; jane.hubert@univ-reims.fr (J.H.); nicolas.borie@univ-reims.fr (N.B.); jh.renault@univ-reims.fr (J.-H.R.); jean-marc.nuzillard@univ-reims.fr (J.-M.N.); dominique.harakat@univ-reims.fr (D.H.); 3 Unité de Recherche Vignes et Vins de Champagne EA 4707, SFR Condorcet FR CNRS 3417, UFR des Sciences Exactes et Naturelles, Université de Reims Champagne-Ardenne, BP 1039, 51687 Reims CEDEX 2, France; eric.courot@univ-reims.fr (E.C.); christophe.clement@univ-reims.fr (C.C.); 4 Centre de Recherche Inserm U866, Université de Bourgogne, 21000 Dijon, France; dominique.delmas@u-bourgogne.fr

**Keywords:** resveratrol, labruscol, melanoma, fibroblasts, cytotoxic activity, bioreactor, *Vitis labrusca* L.

## Abstract

A new resveratrol dimer (**1**) called labruscol, has been purified by centrifugal partition chromatography of a crude ethyl acetate stilbene extract obtained from elicited grapevine cell suspensions of *Vitis labrusca* L. cultured in a 14-liter stirred bioreactor. One dimensional (1D) and two dimensional (2D) nuclear magnetic resonance (NMR) analyses including ^1^H, ^13^C, heteronuclear single-quantum correlation (HSQC), heteronuclear multiple bond correlation (HMBC), and correlation spectroscopy (COSY) as well as high-resolution electrospray ionisation mass spectrometry (HR-ESI-MS) were used to characterize this compound and to unambiguously identify it as a new stilbene dimer, though its relative stereochemistry remained unsolved. Labruscol was recovered as a pure compound (>93%) in sufficient amounts (41 mg) to allow assessment of its biological activity (cell viability, cell invasion and apoptotic activity) on two different cell lines, including one human skin melanoma cancer cell line HT-144 and a healthy human dermal fibroblast (HDF) line. This compound induced almost 100% of cell viability inhibition in the cancer line at a dose of 100 μM within 72 h of treatment. However, at all tested concentrations and treatment times, resveratrol displayed an inhibition of the cancer line viability higher than that of labruscol in the presence of fetal bovine serum. Both compounds also showed differential activities on healthy and cancer cell lines. Finally, labruscol at a concentration of 1.2 μM was shown to reduce cell invasion by 40%, although no similar activity was observed with resveratrol. The cytotoxic activity of this newly-identified dimer is discussed.

## 1. Introduction

Stilbenoids, which are naturally-occurring secondary metabolites widely represented in the plant kingdom, can be divided into monomeric and oligomeric compounds [1]. The most studied stilbene monomer is resveratrol and its biological activity as a phytoalexin in plants or its preventing action against human diseases has already led to a considerable number of works. Though the biological properties of resveratrol are well known, those of the oligomeric resveratrol derivatives are less documented. Most of our knowledge on the activity of stilbene oligomers mainly concerns dimeric structures such as ε-viniferin, pallidol and δ-viniferin. Among dimers, ε-viniferin is the most studied and there are works reporting ε-viniferin to act as an antioxidant [2], anti-inflammatory [3], anticancer [4,5,6,7,8,9], as well as a cardioprotective agent [10]. Limitations in studying the biological properties of resveratrol dimers essentially reside in the difficulty to recover these compounds in large amounts by using conventional plant extraction procedures or chemical synthesis. We have previously described the bioproduction of various phytostilbenes in stirred bioreactors from grapevine cell suspensions [9]. Cultivating grapevine cells in bioreactors in the presence of various defense-inducing compounds, the so-called elicitors [11,12,13,14,15], has indeed been shown to constitute a useful technique for the production of tens to hundreds milligrams of dimeric stilbenes with high purity [9].

Here we report on the characterization of a new stilbene dimer called labruscol (**1**) produced by grapevine cell suspensions of *Vitis labrusca* L. var. Concord as well as the determination of its biological activity (inhibition of cancer cell viability, effect on tumor cell invasion and apoptosis) on the human skin melanoma cancer cell line HT-144, considered to be an aggressive cancer line. Its properties were compared to those of a resveratrol bioproduced from the same cell suspensions.

## 2. Results and Discussion

### 2.1. CPC Purification and Chemical Characterization of Labruscol *(**1**)*

The crude ethyl acetate extract (1.5 g) obtained from the elicited grapevine cell suspensions of *V. labrusca* cultured in a 14-L stirred bioreactor was fractionated by centrifugal partition chromatography (CPC) in a single run of 160 min using a normal phase gradient elution method, including a biphasic solvent system composed of *n*-heptane, ethyl acetate, methanol and water in the ascending mode (see Section 3.3). CPC is a solid support-free separation technique involving the differential partition of solutes between at least two immiscible liquid phases according to their distribution coefficient [16]. The CPC column used here, with a total capacity of 303 mL and 231 partition cells, offers the possibility to collect fractions in sufficient amounts (all > 20 mg) to allow their chemical characterization and determine their biological activity.

**Figure 1:**
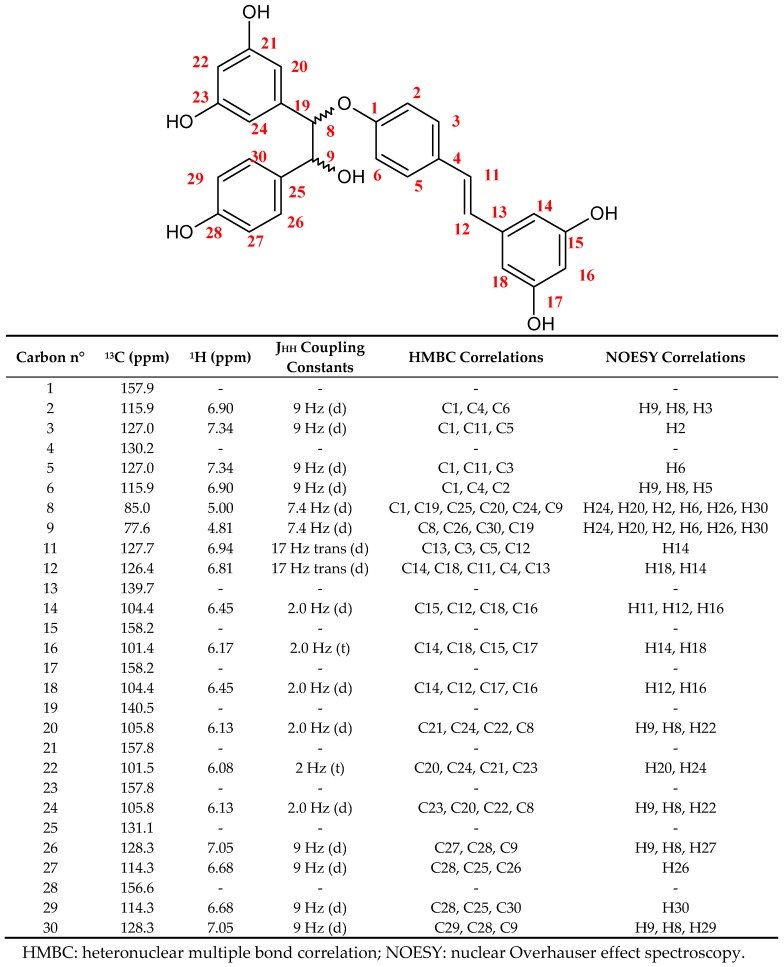
Chemical structure and nuclear magnetic resonance (NMR) data for labruscol (**1**).

All fractions eluted with the organic mobile phase over the gradient contained various stilbene derivatives that together represented 73% of the crude extract injected mass. Besides, the most hydrophilic compounds of the extract, mainly including residual cyclodextrins (used for stilbene elicitation) and culture medium nutrients, were well-retained inside the column (i.e., in the aqueous stationary phase) over the whole CPC experiment. Metabolite identification was performed in the collected fractions by using a ^13^C-nuclear magnetic resonance (NMR)-based dereplication procedure [17,18], revealing *trans*-resveratrol, δ-viniferin, pallidol, ε-viniferin, leachianol F, and leachianol G as the major phytostilbenes bioproduced under the used elicitation conditions [9]. A new resveratrol dimer was also detected in fractions eluted from 115 to 127 min with a purity greater than 93% (41 mg in total). It was not possible to identify its structure during a search in the database, suggesting that the structure of this compound was original. Structure elucidation of this resveratrol dimer, called labruscol (**1**) (Figure 1), was unambiguously achieved by 1D and 2D NMR analyses including ^1^H, ^13^C, heteronuclear single-quantum correlation (HSQC), heteronuclear multiple bond correlation (HMBC), and correlation spectroscopy (COSY). The molecular formula of C_28_H_24_O_7_ was confirmed by high-resolution electrospray ionisation mass spectrometry (HR-ESI-MS) analysis, revealing the molecular ions [M – H]^−^ at *m*/*z* 471 and [2M − H]^−^ at *m*/*z* 943 in the negative ionization mode.

In order to determine the relative stereochemistry of the two stereogenic centers on C8 and C9, a rotating frame Overhauser effect spectroscopy (ROESY) experiment (Figure 1 and Table) together with a conformational optimization of the two putatively relative stereochemistries using a molecular mechanics (MM3*) force field was carried out. Unfortunately, the dihedral angle H8-H9 measured on the two minimized structures corresponding to the 8*R*9*R* (or 8*S*9*S*) or 8*R*9*S* (or 8*S*9*R*) is about 170° in the two cases, which is consistent with the coupling constant *J*_8H-9H_ = 7.4 Hz, but this does not make it possible to conclude on the relative stereochemistry of the dimer (**1**). The interatomic distances were also measured for the two stereoisomers in order to connect them with the nuclear Overhauser effect (NOE) correlations. Once again, the distance differences were not significant and did not allow for making a decision between the two relative configurations. Further studies involving circular dichroism would thus be required to solve the question concerning the absolute configurations of the two stereogenic centers on C8 and C9.

### 2.2. Biological Activity of Resveratrol and Labruscol

**Figure 2:**
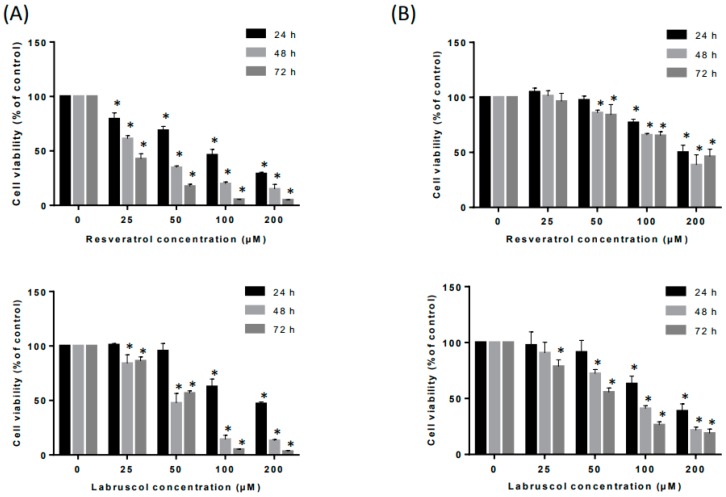

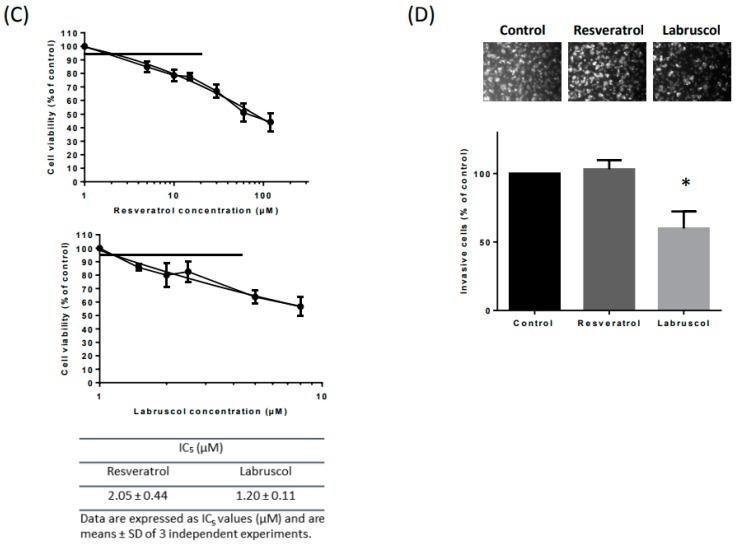
Effects of resveratrol and labruscol on cell viability and cell invasion in HT-144 and HDF cells. (**A**) HT-144 cells were treated with resveratrol and labruscol (0, 25, 50, 100 and 200 µM) in the presence of fetal bovine serum (FBS) for 24, 48 and 72 h before being subjected to an MTT (3-(4,5-dimethyl thiazol-2yl)-2,5-diphenyltetrazolium bromide) assay for cell viability determination. The values represented the means ± standard deviation (SD) of at least three independent experiments; (**B**) Human dermal fibroblast cells were treated with resveratrol and labruscol (0, 25, 50, 100 and 200 µM) for 24, 48 and 72 h before being subjected to an MTT assay for cell viability determination. The values represented the means ± SD of at least three independent experiments; (**C**) Determination of the inhibiting concentration (IC_5_ values of resveratrol and labruscol. HT-144 cells were treated in a medium without FBS with resveratrol (0, 1.5, 10, 15, 30, 60 and 120 µM) and labruscol (0, 1.5, 2, 2.5, 5, 8 µM) for 24 h before being subjected to an MTT assay for IC_5_ determination. The values represented the means ± SD of at least three independent experiments. Straight lines correspond to the IC_5_; (**D**) Determination of cell invasion abilities. Cell invasion was measured using a Boyden chamber for 24 h with a matrigel^®^ coating (25 µg/mL). HT-144 cells were treated in an FBS-free medium with vehicle (ethanol), resveratrol (2 µM) or labruscol (1.2 µM) for 24 h. The invasion abilities of HT-144 cells were quantified by counting the number of cells that invaded the underside of the transwell, as described in the Material and Methods section. The invasive activity of cancer cells was expressed as the mean number of cells that crossed the matrigel. The values represented the means ± SD of at least three independent experiments. * *p* < 0.05, compared with the vehicle group.

The inhibitory effects of the bioproduced resveratrol and labruscol (**1**) at various concentrations (0–200 µM) on the cell viability of the HT-144 melanoma cell line, considered to be an aggressive cancer cell line, for 24, 48 and 72 h were evaluated using an MTT (3-(4,5-dimethyl thiazol-2yl)-2,5-diphenyltetrazolium bromide) assay (Sigma-Aldrich, Saint-Quentin, France) [19] in the presence of fetal bovine serum (FBS). The data are presented in Figure 2A,B. At all tested concentrations and times, resveratrol displayed an inhibition of cancer line viability higher than the dimer, labruscol. Nonetheless, both compounds exerted almost 100% cell viability inhibition at the doses of 100 and 200 μM within 72 h of treatment. In the presence of FBS, the inhibiting concentration (IC_50_) of resveratrol was around 25 μM and that of labruscol around 50 μM, i.e., twice that of resveratrol after 48 h of treatment (Figure 2A,B). Thus, resveratrol displays a higher inhibition of cancer cell viability than its dimers in the presence of FBS, confirming previous results [9].

Figure 2B shows that labruscol induced a higher inhibition of the cell viability of healthy human dermal fibroblasts as compared to resveratrol at all tested concentrations and treatment times. However, this inhibition was markedly lower than the one displayed by this compound on melanoma cells. The two compounds thus showed differential activities on healthy and cancer cell lines and this can lead to interesting applications in cancer therapy [20,21,22]. Without FBS, the determination of the IC_50_ for resveratrol and labruscol led to very different results. With an IC_50_ = 10 μM, labruscol was considerably more effective on cancer cell viability than resveratrol (IC_50_ = 90 μM) (Figure 2C). These data thus confirm that the cytotoxic activity of resveratrol dimers such as ε-viniferin or labruscol is decreased in the presence of FBS, probably due to their interaction with serum proteins [9].

The effects of resveratrol and labruscol on tumor progression, including tumor migration and cell invasion, were tested in vitro on the HT-144 melanoma cell line. To this end we have identified the working concentrations of both resveratrol and labruscol in an FBS-free medium, at which cell viability inhibition does not exceed 5% (IC_5_) (Figure 2C and Table). The MTT assay revealed that the IC_5_ of resveratrol and labruscol were 2 and 1.2 µM, respectively. Concentrations of 1.2 and 2 µM were thus used to test the effect of both compounds on tumor progression. The migration assay was carried out by in vitro wound closure for 0, 12 and 24 h. Results showed that neither resveratrol nor labruscol had effects on tumor cell migration (data not presented). Interestingly, only labruscol was shown to induce a significant inhibition of 40% of cell invasion after 24 h of treatment, while resveratrol had no effect on cell invasion at the same concentration (Figure 2D). Although there are previous works reporting on the capacity of resveratrol to inhibit cell migration and invasion in other cell lines, this was only observed at concentrations higher than those used in this study (between 5 and 50 µM) [23,24,25,26].

The data presented here clearly show that resveratrol and the newly identified resveratrol dimer, labruscol, have interesting antiproliferative activities. Indeed, both compounds exert a marked inhibition of the cell viability of melanoma cells compared to healthy cells, although resveratrol displays a reduced impact on the cell viability of normal cells.

This tumor specificity has also been reported in the case of other phytostilbenes [6]. α-viniferin and *trans*-miyabenol C, two trimers of resveratrol, for example, were shown to exert a higher inhibiting activity on human colorectal carcinoma cells than on healthy colorectal lines [27]. The same activity was described for gnetin H, a resveratrol trimer in lung and breast carcinoma compared to healthy lines [28]. Due to the difficulty of obtaining resveratrol oligomers in sufficient amounts as pure compounds, there are only a few reports of other resveratrol dimers having similar inhibitory effects on the cell viability of cancer cells. These previously published results mainly concerned the effects of pallidol on human colorectal carcinoma cell lines (HCT1116, HT-29 and Caco-2) [27] and those of ε-viniferin on murine leukemia cell lines (P-388) [7], human oral squamous carcinoma cell lines (HL-60) [6], lymphoid and myeloid cell lines (U266, RPMI-8226, U937, K562, Jurkat) [8]. Pallidol and ε-viniferin were both reported to have inhibitory activities on the cell growth of two melanoma skin cancer cell lines, HT-144 and SKMEL-28 [9]. ε-viniferin also displays antiproliferative effects in human hepatocyte derived Hep G2 cells [4].

**Figure 3:**
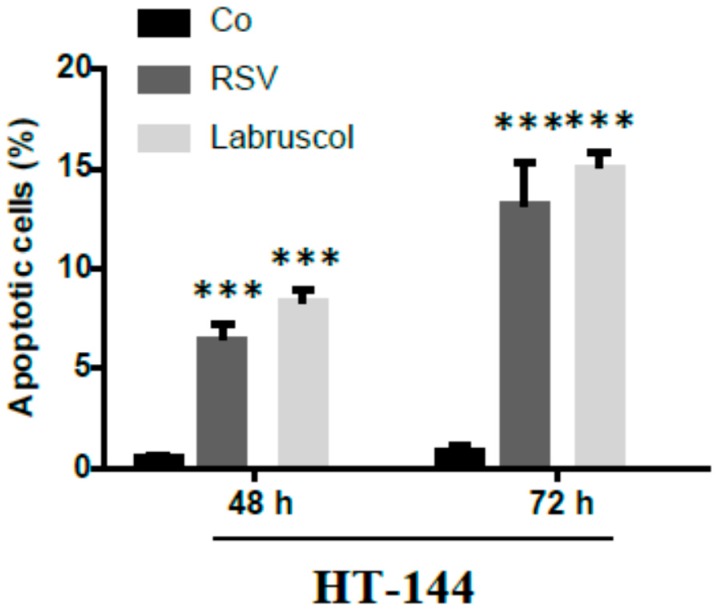
Apoptosis induction by resveratrol and labruscol in human skin melanoma cancer cells. Cells were treated during 48 h or 72 h with 50 μM resveratrol or labruscol. Apoptosis was assessed by staining (control cells or cells treated with resveratrol or labruscol) with 1 μg/mL Hoechst 33342. Co: control cells; RSV: resveratrol-treated cells; Labruscol: labruscol-treated cells. The values represented the means ± SD of at least 3 independent experiments. *** *p* < 0.05, compared with the control group.

Labruscol and resveratrol (50 μM) were also shown to induce apoptosis in the HT-144 cancer cell line reaching 15% of apoptotic cells for both compounds within 72 h treatment (Figure 3).

This suggests that apoptosis induction in cancer cells could be a possible mechanism for the antiproliferative effects of labruscol, as reported in previous studies for resveratrol and related metabolites [29]. On the other hand, a transient senescence activity has already been described for resveratrol in human metastatic colon cancer cells [30]. As the chemical structure of labruscol is analogous to that of resveratrol (presence of a trans-resveratrol moiety), one can assume that labruscol can also inhibit tumor cell growth via a senescence induction pathway [30]. Further works are thus needed to characterize the mechanisms implied in the cytototoxic activities of labruscol reported in the present study.

In sum, the results obtained here with the aggressive cancer cell line HT-144 reveal the antiproliferative activity of a newly characterized resveratrol dimer, labruscol. Moreover, at the very low concentration of 1.2 µM (IC_5_), this compound has shown a 40% inhibition of cancer cell invasion, a property not displayed by resveratrol. It thus seems that labruscol possesses complementary properties of resveratrol in particular regarding cell invasion. One can thus suggest that labruscol could be used in combination with resveratrol to improve its antiproliferative capacities. Other studies have indeed already shown a greater efficiency of mixtures of stilbenes compared to resveratrol alone [4,31,32]. As stilbene derivatives generally do not display high cytotoxic activity, the biological effects of labruscol could thus be assessed in the context of multi-drug resistance, where this compound alone or in combination can actively participate to cell resensitisation by therapeutic agents, helping in the reduction of their doses and their toxicities [29].

## 3. Materials and Methods

### 3.1. Chemicals, Reagents and Materials

Methanol (MeOH), ethyl acetate (EtOAc), and *n*-heptane (Hept) were purchased from Carlo ErbaReactifs SDS (Val de Reuil, France). Deuterated methanol (methanol-*d4*) was purchased from Sigma-Aldrich (Saint-Quentin, France). Deionized water was used to prepare all aqueous solutions.

### 3.2. Cultures in Bioreactor and Elicitation of Stilbene Production

Cell suspensions of *Vitis labrusca* L. var. Concord were cultured in a 14 L tank of a stirred bioreactor Bioflo 3000 (New Brunswick Scientific, Edison, New York, NY, USA) containing 10 L (final volume) of B5 medium [33]. The agitation (two marine turbines) was set to 50 rpm and the aeration rate maintained at 0.025 vvm. All other conditions were as previously described [9]. Stilbene production was induced by the use of two elicitors. The first elicitor was a β-cyclodextrin Kleptose^®^ (Roquette, Lestrem, France). The second elicitor was methyljasmonate. These two elicitors were added to the cell cultures as previously reported [9].

### 3.3. Extraction of Total Stilbenes from the Culture Medium and CPC Purification of Labruscol

The whole culture medium (10 L) was filtered under reduced pressure and stored at −20 °C until use. A crude stilbene extract (3.1 g) was obtained from 5 L of the filtered culture medium by performing three successive extractions with ethyl acetate (3 × 2 L) in a separatory funnel followed by solvent elimination under vacuum at 40 °C.

Centrifugal partition chromatography (CPC) was carried out on a lab-scale FCPE300^®^ column of 303 mL capacity (Rousselet Robatel Kromaton, Annonay, France) containing seven circular partition disks, engraved with a total of 231 oval partition twin-cells (~1 mL per twin-cell) and connected to a KNAUER Preparative 1800 V7115 pump (Berlin, Germany). The system was coupled to a UVD 170S detector set at 210, 254, 280, and 366 nm (Dionex, Sunnivale, CA, USA). Fractions were collected by a Pharmacia Superfrac collector (Uppsala, Sweden).

Labruscol was purified by using a gradient elution method as described previously [9]. Fractions were collected every minute, spotted on Merck thin layer chromatography (TLC) plates coated with silica gel 60 F254 and developed with chloroform/ethyl acetate/formic acid (6:4:1, *v*/*v*/*v*). After UV detection at 254 nm, the plates were sprayed with vanillin–sulfuric acid and heated to 100 °C for 5 min. Labruscol was detected as a pink stain with a retention factor of 0.67 in these TLC conditions.

### 3.4. Structural Elucidation of Labruscol

NMR analyses were performed in methanol-*d*_4_ at 298 K on a Bruker Avance AVIII-600 spectrometer (Karlsruhe, Germany) equipped with a cryoprobe optimized for ^1^H detection and with cooled ^1^H, ^13^C and ^2^H coils and preamplifiers. 1D and 2D NMR spectra (^1^H, ^13^C, HSQC, HMBC, NOESY and COSY) were recorded using standard Bruker pulse programs (Bruker, Karlsruhe, Germany). An aliquot of labruscol was also solubilized in MeOH and directly infused in a quadrupole time-of-flight hybrid mass spectrometer (QTOF micro^®^, Waters, Manchester, UK) equipped with an electrospray source. The mass range of the instrument was set at *m*/*z* 100–1200 and scan duration was set at 2 s in the negative ion mode. The capillary voltage was 3000 V, the cone voltage was 35 V, and the temperature was 80 °C.

Conformational analysis to find the lowest energy conformers for the two possible relative stereochemistry *RR* or *RS* using the force field MM3* in octanol were done with Maestro module of the Schrödinger Suite, version 10.5.014 (Shrödinger Software, San Diego, CA, USA). No constraints were applied. Conformational search using mixed torsional/low-mode sampling was used. The number of separate conformers generated was 1000, with a maximum of 100 unique structures to be saved for each rotatable bond. A 21 kJ/mol energy cutoff was used to remove the higher energy conformers. A conformer was considered redundant and subsequently eliminated if its maximum atom deviation from an already-identified conformer was less than 0.5 Å. All conformers were subjected to further minimization using the Powell–Reeves conjugate gradient (PRCG) method for a maximum of 2500 steps, by using a convergence threshold of 0.05.

### 3.5. Biological Tests

#### 3.5.1. Cell Cultures

The HT-144 cell line used in this study derived from human melanoma and was obtained from the American Tissue Culture Collection (ATCC). Cells were grown in a McCoy’s 5a modified medium supplemented with 10% (*v*/*v*) FBS and 1% (*v*/*v*) antibiotic (penicillin, streptomycin) at 37 °C in a humidified atmosphere of 5% CO_2_. Human dermal fibroblasts (HDF) were isolated from skin biopsies of healthy subjects. Their culture conditions were as described previously [9].

#### 3.5.2. Experimental Treatments

The two cell types were seeded during 24 h before to be treated in triplicate wells with resveratrol and labruscol at different concentrations and treatment times. Compounds were dissolved in ethanol at a 5 × 10^−2^ M final concentration and stored at −20 °C. Compounds were diluted in culture media, with or without FBS, to the desired final concentration. All control and treated cells received a maximal volume of 0.1% (*v*/*v*) of ethanol.

#### 3.5.3. Cell Viability Assay

Cell viability was examined by MTT [3-(4,5-dimethyl thiazol-2yl)-2,5-diphenyltetrazolium bromide] assay (Sigma-Aldrich, Saint-Quentin, France) [19] as previously described [9]. The IC_5_ for the bioproduced stilbenes, resveratrol and labruscol, was defined as the concentration producing 5% decrease in cell growth.

#### 3.5.4. In Vitro Wound Closure

HT-144 cells (2 × 10^5^ cells/well) were plated in six-well plates for 24 h, wounded by scratching with a pipette tip, incubated in McCoy’s 5a modified medium without FBS, and treated or not with resveratrol and labruscol at the same concentration, which did not exceed their IC_5_, 1.2 µM for 0, 12 and 24 h. The cells were photographed, in three same fields by well, at 0, 12 and 24 h using a phase-contrast microscope (100×).

#### 3.5.5. In Vitro Invasion Assays

The invasive potential of tumor cells was examined using modified Boyden chambers (6.5 mm diameter and 8 µm pore) (Greiner Bio-One, Les Ulis, France) according to the manufacturer’s instructions. Briefly, HT-144 cells were suspended in a serum free McCoy’s 5a modified medium and 100 µL of the cell suspension (2 × 10^4^ cells) were seeded onto the upper compartment of the Transwell coated with 25 µg of Matrigel (Corning Life Sciences, Corning, NY, USA). Cells were treated, or not, 1 h after being seeded with resveratrol and labruscol at 1.2 µM respectively for 24 h. In the lower compartment, 800 µL of the McCoy’s 5a modified medium containing 10% FBS were added 2 h after treatment and used as chemoattractant. After 24 h, cells were fixed with methanol, being the non-invading cells remaining on the upper side of the filter, scrapped off. Invading cells on the lower side of the filter were stained with Hoechst 33342 (Sigma-Aldrich, Saint-Quentin, France). Invading cells were observed with a fluorescence microscope and counted in five fields at 100× magnification. The invasive activity of cancer cells was expressed as the mean number of cells that crossed the Matrigel.

#### 3.5.6. Apoptosis Identification

Apoptosis was identified by staining the nuclear chromatin of trypsinized cells (controls and resveratrol or labruscol-treated cells) with 1 μg/mL Hoechst 33342 (Sigma Aldrich, Saint Quentin, France) for 15 min at 37 °C. The percentage of apoptotic cells was determined by the analysis of 300 cells from randomly fields for each treatment.

### 3.6. Statistical Analysis

The data were expressed as the mean ± standard deviation (SD) of three independent experiments. Each experiment was performed in triplicate. The significance of differences was established with the Student’s *t*-test.
